# A Theoretical Prediction of the Antioxidant and Electronic Properties of Epicatechin, Procyanidin B2, Procyanidin, C1 and Cinnamtannin A2

**DOI:** 10.3390/molecules31111876

**Published:** 2026-05-29

**Authors:** Boleslaw T. Karwowski

**Affiliations:** Nucleic Acids Damage Laboratory, Food Science Department, Faculty of Pharmacy, Medical University of Lodz, ul. Muszynskiego 1, 90-151 Lodz, Poland; boleslaw.karwowski@umed.lodz.pl

**Keywords:** (−)-epicatechin, procyanidin B2, procyanidin C1, cinnamtannin A2, ionisation potential, electron affinity, global reactivity descriptors, Fukui reactivity index, density functional theory

## Abstract

During food intake, small amounts of antioxidants are absorbed and distributed to the extracellular matrix, from which they are made available to all types of cells. They protect against various free radicals generated in the extracellular and intracellular environments. They also protect against ionising radiation or UV directly. Some of the most abundant antioxidants in food are the proanthocyanidins, a form of condensed tannin found in tea, cocoa, and grape seeds. They also bestow various other health benefits as apoptosis inducers. The present study examines the vertical and adiabatic ionisation potentials and electron affinities of flavan, (−)-epicatechin, procyanidin B2, procyanidin C1, and cinnamtannin A2, and discusses the influence of non-equilibrated solvent–solute interactions on their electronic properties. The analysis employs the M06-2x/aug-cc-pVTZ//M06-2x/6-31++G** level of theory in the aqueous phase. Procyanidin C1 was found to have the lowest ionisation potential (6.08 eV) and the highest adiabatic electron affinity (1.15 eV); also, all (−)-epicatechin derivatives demonstrated a lower IP than guanine (6.42 eV), suggesting a potential genome-protective effect. These findings were confirmed by the global reactivity descriptor, the Fukui reactivity index, and the spin density distribution. The theoretical results presented here support the experimental results which predict that nutrients can help maintain a delicate redox balance which is crucial for the extra- and intracellular matrix.

## 1. Introduction

The human body contains approximately 10^14^ cells connected by the extracellular matrix (ECM) [1]. In addition to acting as an extracellular glue, the ECM also transports proteins, sugars, and fatty acids, as well as vitamins and minerals, required for cell growth and development [2]. However, it can also serve as the environment for xenobiotics and other harmful factors, such as cell metabolic products, reactive oxygen/nitrogen species and aldehydes, which interact with the cell membrane, or with various intracellular proteins and nucleic acids after absorption [3,4]. An increase in the activity of these factors can cause, e.g., ferroptosis, apoptosis, necrosis, inflammatory processes, or mutagenesis, and ultimately carcinogenesis [5,6]. In response, cells have developed multistage protective systems. These can be divided into non-enzymatic and enzymatic types, which deactivate free radicals (e.g., reactive oxygen/nitrogen species: RO/NS) and reduce oxidative stress [7]. The first line of the cellular defence system consists of low-molecular-weight molecules known as antioxidants [8]. Many antioxidants are obtained from food and, despite being present in small amounts, can slow, terminate, or manage oxidation processes. If the cellular antioxidant pool is depleted, the delicate balance is disrupted, leading to harmful processes such as lipid peroxidation, DNA damage, or protein dysfunction [9]. If the resulting lesions are not repaired by systems such as the DNA Damage Response (DDR), the cell can undergo ageing, death, or mutagenesis/carcinogenesis [9]. Therefore, protecting the external and internal cellular environment against harmful factors is crucial not only for maintaining genetic information stability, but also for cell survival.

Among the variety of natural compounds present in food, the most abundant are probably the flavonoids, which belong to the second class of metabolites. More than 9000 different compounds are currently known [10]. These compounds have shown a wide range of therapeutic antioxidant, anti-inflammatory, anticarcinogenic, and antimutagenic activities, albeit with prooxidant functions observed in some cases [11]. Due to their bioactivity, these molecules have become the subject of numerous clinical studies; many of which address their cytoprotective and anticancer activities. Indeed, 20 million new cancer incidents were recorded in the year 2022, with a mortality rate of 42%, and 35.5 million new cases are predicted in 2050 [12]. As such, there is increasing demand for new therapies that avoid the serious side effects typical of chemo- or radiotherapy.

**Figure 1:**
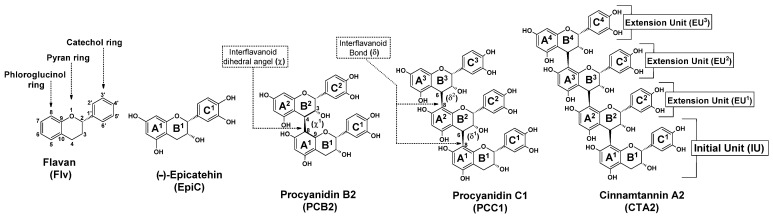
Graphical representation of flavan leading structure and selected antioxidants: (−)-epicatechin, procyanidin B2, procyanidin C1, cinnamtannin A2 with suitable atom ring numbering. Also, interflavonoid bond δ (C4–C8) and dihedral angle χ (C3–C4–C8–C9) have been indicated.

Recently, attention has been paid to procyanidins, the most abundant compounds in the tannin family [13]. These molecules are condensed derivatives of flavan, such as (−)-epicatechin or (+)-catechin. They can exist as dimers (procyanidin A and B), trimers (procyanidin C), and tetramers (arecatannin A2 and cinnamtannin A2), see Figure 1 [14]. Despite possessing differing degrees of condensation, these molecules exhibit wide therapeutic potential, including anticancer, anti- and prooxidant, proinflammatory, apoptotic, chemotherapeutic, chemosensitising, senomorphic, and senotherapeutic effects [15].

Although the biological activity of condensed tannins has been well studied, no systematic investigation of their electronic properties has been carried out, especially with regard to equilibrated or non-equilibrated solvent–solute interactions [16,17,18,19]. In particular, no comparative density functional theory (DFT) studies have been performed to determine the anti- or prooxidative properties of condensed tannins [20,21]. Such findings could elucidate the influence of antioxidants on charge transfer between macromolecules present in the cytosol and on mutual communication between the extracellular matrix (ECM) and the intracellular environment. They could also cast light on their potential to inhibit undesirable processes, such as carcinogenesis and ageing, and support more desirable ones such as apoptosis.

Previous theoretical studies on condensed tannins have mainly focused on the antioxidant mechanisms of simple flavonoids and monomeric polyphenols, bond dissociation enthalpies (BDE), proton affinities (PA), HOMO/LUMO analysis, and selected conformational properties of procyanidin dimers and trimers [14,16,22,23,24]. Mendoza-Wilson et al. examine the relationship between structure and radical scavenging in procyanidins, mainly in the context of thermodynamic antioxidant descriptors and HOMO/LUMO properties [19]. In contrast, Tarascou et al. focus predominantly on conformational behaviour using interflavonoid dihedral-angle NMR analysis, together with molecular modelling approaches [24]. Most density theoretical (DFT) studies of flavonoids have concentrated on hydrogen atom transfer (HAT), sequential electron-transfer proton-transfer (SET-PT), and sequential proton-loss electron-transfer (SPL-ET) antioxidant mechanisms [16,25,26]. Very little, if any, research has systematically addressed the influence of non-equilibrated versus equilibrated solvent–solute interactions on condensed tannin radical formation, vertical and adiabatic ionisation/electron attachment processes, or the charge-dependent conformational behaviour of PCB2, PCC1, and CTA2, neither has it examined solvent-dependent spin-density redistribution in radical cationic and radical anionic states. Therefore, the present article takes a novel approach in that it applies a complex analysis based on various parameters: Vertical and adiabatic ionisation potentials/electron affinities, non-equilibrated and equilibrated solvent-relaxation effects, global reactivity descriptors, Fukui reactivity indices, and spin-density distributions. It also examines the charge-induced conformational changes taking place in a homologous series of condensed tannins, ranging from monomeric (−)-epicatechin to tetrameric cinnamtannin A2, in the aqueous phase at the M06-2X/aug-cc-pVTZ level of theory.

## 2. Results

The antioxidant properties of the molecules can be discussed in terms of three mechanisms: hydrogen atom transfer, single electron transfer with accompanying proton transfer, and sequential proton-loss electron-transfer [25,26]. All of the above depend on the electronic properties, that is, the ionisation potential (IP) and electron affinity (EA), which describe the ability of a molecule to eject or accept an electron. These can be calculated in the vertical mode according to the Koopmans’ or Janak’s theorem, which exploits the energies of highest occupied (HOMO) and lowest unoccupied molecular orbitals (LUMO) of the neutral ground state geometry [27,28]. The M06-2X functional has been extensively benchmarked for the thermochemistry, radical reactions, non-covalent interactions, and antioxidant-related electronic properties of polyphenolic compounds. Recent DFT benchmark studies on antioxidant-related descriptors have found that M06-2X protocols provide good performance for the bond dissociation enthalpies, ionisation potentials, proton affinities, and electron-transfer parameters of phenolic antioxidants [29,30,31,32,33]. Therefore, the M06-2X hybrid-meta functional was chosen for the current research based on radical cationic and radical anionic states. Also, M06-2X has demonstrated good agreement with G4 calculations for O–H bond dissociation energy and electronic properties in comparative studies on flavonoids [34,35]. The 6-31++G** basis set was used for geometry optimisation, which provided a good balance between the cost and accuracy of the computations. Additionally, diffuse functions are essential for obtaining a correct geometry description for charged radicals. Single-point energy calculations were subsequently refined using the larger aug-cc-pVTZ basis set, which is widely used to calculate ionisation potential and electron affinity due to the inclusion of extended diffuse and polarisation functions [36,37,38,39]. Instead of a microsolvation state or periodic box models with random water molecule distribution the conductor-like polarisable continuum model, the C-PCM model combined with water dielectric constant (ε = 78.4) was selected for anion/cation radical formation. The conductor-like polarisable continuum model enables the efficient treatment of solvent polarisation during vertical and adiabatic charge transfer, including non-equilibrated and equilibrated solvent–solute interactions. Such models are commonly used in studies of antioxidant molecules, and electron/hole transfer reactions in aqueous (condensed) environments or phases. Moreover, previous benchmarks have demonstrated that C-PCM reproduces the aqueous solvation free energies and electronic properties of neutral and ionic organic molecules with good accuracy and numerical stability [40,41].

### 2.1. Structural Differences Between the Adiabatic Neutral, Anion, and Cation States

The spatial structures of the neutral and radical cation/anion forms of the molecules presented in Figure 1 were optimised at the M06-2x/6-31++G** level of theory in the aqueous phase, using the C-PCM (conductor-like polarisable continuum model) solvation model [40,42,43]. To confirm the ground state of the obtained geometries, the Hessian matrix was calculated using the same functional and basis set in the aqueous phase [44]. For all discussed moieties, no imaginary frequencies have been found. The initial spatial geometries of all discussed compounds were constructed based on previously reported structures [31,34,35]. The variation in the interflavonoid dihedral angle χ (C3–C4–C8–C9) was considered, as noted previously for both the compact and extended conformations of PCB2, PCC1, and CTA2. The structures of all discussed molecules were optimised to the ground state at the M06-2X/6-31++G** level of theory in the aqueous phase. None of the obtained structures demonstrated any imaginary frequency in their neutral, anion/cation radical state. The same level of theory was used to calculate the frequency. The above confirms that all reported spatial geometries correspond to local minima. It should be pointed out that condensed tannins possess a large number of conformational states. However, previous research has found the main electronic descriptors of procyanidins, including HOMO/LUMO distribution and antioxidant-related reactivity, to be primarily governed by the catechol/phloroglucinol electronic structure and delocalisation effects, rather than by conformational fluctuations [19]. Hence, although quantitative differences in ionisation potential, electron affinity, Fukui reactivity index, or spin density distribution may occur between conformers, the overall trends observed in the present study are expected to remain qualitatively preserved; conformational flexibility may however contribute to minor variations in the calculated descriptors.

To elucidate the structural changes induced by electron addition or loss in the system, the RMSD (Root Mean Square Deviation) was calculated [45]. The obtained results indicate that the most sensitive to electronic changes was PCC1. The RMSD was found to be 0.3514 [Å^2^] for the neutral versus radical cation, but more than 10 times higher, i.e., 3.8027 [Å^2^], for the anion. Contrary to the above, the higher structural stability, i.e., lower RMSD, was denoted for EpiC: 0.1888 and 0.2758, anion and cation versus neutral, respectively (Table 1). It should be noted that the structures of the condensed tannins PCC1 and CTA2 exhibit higher resistance to electron loss than the (−)-epicatechin dimer PCB2 (Table 1). The presence of an additional flavan-3-ol subunit leads to condensed tannins, which, together with the increased number, reduce the structural fluctuations forced by additional electrons. Importantly, the pore dimension of gut epithelium, which allows for nutrient absorption, was estimated as 16–26 Å [22,46,47]. Therefore, the length and molecular volume (M_V_) of the compounds in their neutral, radical anion and cation forms were considered in the analysis.

The variability of M_V_ for the leading structure (Flavan) is presented in Table 1. As expected, the adoption of an electron increases the volume of the molecule, while electron loss decreases it. Conversely, extending the structure by hydroxyl group addition ((−)-epicatechin) or tannin condensation up to three units (PCB2 or PCC1) results in the radical cation exhibiting a higher M_V_ value than the neutral molecule, while the radical anion forms a lower one. Surprisingly, the subsequent addition of an epicatechin unit abolishes this trend and returns to the pattern observed for flavan. Therefore, the appearance of an additional charge (positive or negative) causes structural changes. These findings are in close agreement with the RMSD analysis. Therefore, the analysis includes a comparison of the molecular lengths of its neutral, anion, and cation forms.

Based on the calculated distances between proximal and distal protons, the tannin geometry was found to shorten in the following order: neutral > radical anion > radical cation. However, procyanidin C1 demonstrated a significant decrease in length by ~1 Å after electron addition. It should be noted that all molecular lengths of the discussed compound range from 9.47 to 21.15 Å. The above analysis shows that, regardless of the charged form, the molecule is smaller than the gut epithelial pore. It can therefore be absorbed from the intestinal lumen into the ECM. The estimated pore diameter range of approximately 16–26 Å was added, based on the studies of Fine et al., Buckley et al., and Mendoza-Wilson et al. [22,46,47]. It should be noted that these compounds are not affected by the addition or loss of extra electrons in the same manner because the resulting structural response depends strongly on charge delocalisation, interflavonoid interactions, and conformational relaxation within the system. In particular: (a) electron addition increased molecular volume in flavan, while electron loss reduced it, (b) whereas radical cation formation produced a slight increase in molecular volume and structural rearrangement for PCB2 and PCC1. This difference may result from the differences in charge distribution patterns and the solvent-induced conformational relaxation occurring in highly hydroxylated systems’ (−)-epicatechin derivatives.

The simplest condensed tannin, PCB2, can exist as a compact and extended conformation, depending on whether the dihedral angle (χ) between the condensed epicatechin units, i.e., C3–C4–C8–C9, is positive or negative (Figure 1) [23]. The conformation analysis found that PCB2 adopted the compact conformation, indicated by a positive χ^1^ value in the range 88.4–89.3°, irrespective of charge (Table 2). Surprisingly, in PCC1, the χ^1^ between the initial (IU) and the extended units (EU^1^) was found to be negative, i.e., with a value between −88.4° and −89.8°, and to indicate an extended conformation in all cases. Contrary to χ^2^ between EU^1^ and EU^2^, their neutral and radical cation forms demonstrated a compact conformation, while additional electron adoption resulted in an extended one (Table 2). The used terminology (compact and extended conformation) is consistent with previous NMR and computational studies of procyanidins by Tarascou et al., who propose that the compact or extended arrangement of neighbouring flavan-3-ol units are determined by interflavonoid dihedral angles in aqueous environments. Similar conformational classifications were also discussed by Mendoza-Wilson et al. [16,34,35]. Hence, PCC1 structure is highly charge dependent. Regarding CTA2, the addition of EU^4^ to PCC1 (Figure 1) resulted in the following conformations: IU/EU^1^ extended, EU^1^/EU^2^ compact, and EU^2^/EU^3^ extended. It should be noted that previous studies have found compact conformation to predominate in the aqueous phase [23,24]. The mutual position of (−)-epicatechin subunits is dependent on interflavonoid dihedral angle (χ) fluctuation, which is inextricably related to the interflavonoid bond (δ) between C4 and C8 (Figure 1). The δ-bond analysis found it to be resistant to charge fluctuation: In the case of PCB2 and PCC1, δ^1^ and δ^2^ were 1.52 Å in length (Table 2). For CTA2, electron loss leads to a decrease in δ^2^ and δ^3^ lengths from 1.52 Å in the neutral form to 1.50 Å and 1.51 Å, respectively. In cinnamtannin A2, electron adoption causes δ^3^ to shorten by 0.01 Å compared to the neutral form (Table 2).

### 2.2. The Electronic Properties of the Condensed Tannin Derivative

**Figure 2:**
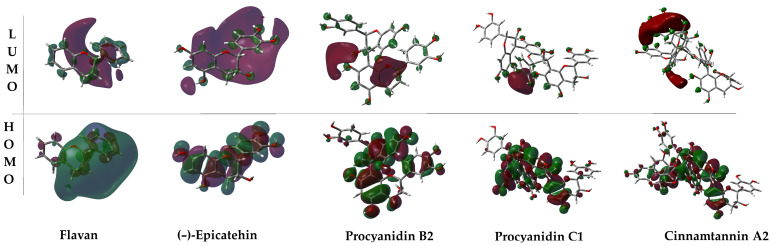
The graphical representation of HOMO and LUMO calculated on the M06-2x/aug-cc-pVTZ level of theory in the aqueous phase of antioxidant neutral ground state geometries.

According to Halliwell, antioxidants can decrease localised oxygen concentrations, prevent chain initiation by scavenging initiating radicals, bind catalysts such as metal ions to prevent radical formation, decompose peroxides, and disrupt the chain reaction, thus preventing continued hydrogen abstraction by active radicals [48]. These properties depend on the ability of the molecule to undergo electron loss or gain, which are directly related to the HOMO and LUMO energies (*E^H^* and *E^L^* respectively) (Figure 2). It is thus possible to estimate the vertical ionisation potential and vertical electron affinity in accordance with Koopmans’ theorem. Among all the analysed molecules, the lowest HOMO energy was noted for CTA2, and the lowest LUMO energy for cinnamtannin A2. Our findings indicate that Δ*E^H-L^* demonstrated the following descending order: Flv > EpiC > PCB2 > PCC1> CTA2 (Table 3). Hence, the smallest gap between HOMO and LUMO was calculated for CTA2 (6.77 eV), which predicts that it can form the most stable radical. It therefore exhibits good antioxidant properties, along with lower kinetic stability and higher chemical reactivity. It should be noted that all biologically active compounds, including antioxidants, operate in aqueous environments. Therefore, the solvent–solute interaction should be taken into consideration when determining the electronic properties in non-equilibrated (NE) and equilibrated (EQ) modes. The loss of an electron from the valence orbital leads to vertical radical cation formation; according to the Oppenheimer rule, subsequent nuclear relaxation causes the adiabatic radical cation state [49]. The above corresponds to vertical (VIP) and adiabatic ionisation potentials (AIP), respectively. In the first step of the ionisation process, electron loss occurs without solvent shell relaxation (NE), followed by subsequent environmental molecule relaxation (EQ), quantified as ^NE^VIP and ^EQ^VIP. This change can predict the rate of radical species neutralisation, i.e., antioxidant potential.

In condensed tannins, the vertical ionisation potential decreases as the number of flavans increases (Table 3). In addition, ^NE^VIP, and ^EQ^VIP were found to decrease in the following order: Flv > EpiC > PCB2 > PCC1 > CTA2. It should be pointed out that the values of the vertical ionisation potential calculated for the non-equilibrated solvent–solute interaction were found in the range between 7.53 and 7.05 eV, and these values decreased by 0.7–1 eV after solvent relaxation. Nuclear relaxation causes an adiabatic radical state, which can be quantified by AIP. The following AIP order was observed: Flv > EpiC > CTA2 > PCB2 > PCC1 (Table 3). Surprisingly, the AIP of PCC1 was found to be 0.02 eV lower compared to the flavan dimer (PCB2), and 0.03 eV lower than the tetramer (CTA2). These small differences in AIP suggest that the condensed tannins may possess equal antioxidant properties after the relaxation of their radical cation spatial structures.

In contrast, the ability of the molecule to accept an electron and form a radical anion is represented by electron affinity (AE). Similarly to the IP, the EA can be discussed in the NE and EQ vertical modes, and after spatial-geometry relaxation by adiabatic electron affinity (AEA). A negative ^NE^VAE was noted for monomers, i.e., Flv and EpiC (Table 3), indicating that electron attachment is not preferred. Surprisingly, for the condensed tannins, this parameter has a positive value, making the initial step of the vertical radical anion formation reasonable. The highest ^NE^VEA was calculated for CTA2 (0.12 eV). For the subsequent solvent–solute equilibration step, at each point, the positive ^NE^VEA has been found in the following order: Flv > EpiC > PCB2 > PCC1 > CTA2. Further nuclear relaxation leads to adiabatic radical formation; the resulting AEA values were as follows (in eV): EpiC(1.18) > PCC1(1.15) > Flv(1.05) > CTA2(0.84) > PCB2(0.63). It should be noted that Guanine (Gua), which has the lowest IP among the canonical nucleobases, exhibited a higher ionisation potential than the discussed antioxidants (Table 3) [50]. Moreover, the ^NE^VEA and ^EQ^VEA were lower than those for monomeric and condensed tannins. Surprisingly, the AEA of Gua was higher than that found for PCB2 and CTA2.

### 2.3. The Antioxidant Global Reactivity Descriptors

As mentioned in paragraph 2.2, the stability and reactivity of a molecule can be predicted from its Δ*E^H-L^*, with its stability increasing with the energy gap. Also, the vertical ionisation potential (VIP) and electron affinity (VEA) can be estimated from the HOMO/LUMO energy according to Koopmans’ theorem. In addition, the VIP, VEA, and adiabatic IP and EA can be calculated from the energy of the suitable ground state; the vertical parameters can also be calculated for the different molecular states (vertical NE/EQ) to show the influence of solvent–solute interaction [51].

Therefore, based on the above, the following global reactivity descriptors can be calculated in the following four modes: chemical hardness (*η* = 0.5[VEA − VIP]) and softness (*S* = 1/*η*), global electrophilicity index (ω = μ2/2η), and electronic chemical potential (*μ* = 0.5[VEA + VIP]). These are presented in Table 4 [52]. Among all the molecules under discussion, flavan demonstrates the highest *η* values according to Koopmans’ Theorem (Koopmans’ Th.), vertical NE, vertical EQ and adiabatic modes, i.e., 3.74, 3.79, 2.78, and 2.59 eV, respectively, which predict their highest stability. At the other end of the hardness scale lies CTA2 (3.39, 3.47, 2.77, 2.64 eV) which is more reactive than the others. Our findings indicate that *η* decreases in the following order among the investigated tannin derivatives: EpiC > PCB2 > PCC1 > CTA2. According to Pérez’s prediction, molecules can be regarded as strong electrophiles when the global electrophilicity index (*ω*) adopts a value that is above 1.5 eV, weak below 0.8 eV, and moderate between 1.5 and 0.8 eV [52,53,54]. All considered antioxidants adopt a value in the range from 1.80 eV to 2.70 eV, independent of the calculation mode *ω*. Therefore, all the discussed antioxidants appear to be strong electrophiles (Table 4). The highest value was found for the tannin monomer, i.e., EpiC (2.70 eV), calculated in adiabatic mode. The electronic chemical potential (*μ*) reflects how likely a system is to either gain or lose electrons. A high negative value indicates that the molecule is an effective electron acceptor, while a less negative value implies a stronger electron donor. In these studies, among the discussed flavan derivatives, the following order of electronic chemical potential was found: Flv > EpiC > PCB2~PCC1 > CTA2. Similar results were obtained for VIP and VEA according to Koopmans’ theorem (Table 3). The results obtained for *η*, *S*, *μ*, and *ω* using vertical non-equilibrated, vertical equilibrate, and adiabatic states indicate in all cases, decreases in chemical hardness are accompanied by system relaxation and increases in softness. Additionally, in the discussed antioxidant system, a decrease in *η* with a subsequent rise in *ω* was observed in the following order of states: vertical NE → vertical EQ → adiabatic (Table 4).

### 2.4. The Fukui Reactivity Index of Flavan, (−)-Epicatechin, and Condensed Tannins

Due to the considerable variety of reactive molecules present in the ECM and cytosol, including electrophiles, nucleophiles, and radicals, the susceptibility of atoms within antioxidant structures merits particular interest. A suitable indicator for this purpose is the Fukui reactivity index, which provides information about the reactivity of atoms in the molecule [55]. Therefore, the atom nucleophilic (*f*_k_^+^), electrophilic (*f*_k_^−^), and radical (*f*_k_^0^) attack susceptibility factors, together with the dual descriptor (Δ*f*_k_), were calculated thus:

*f_k_*^+^ = *q_k_*(*N* − 1) − *q_k_*(*N*), (1)

*f_k_*^−^ = *q_k_*(*N* + 1) − *q_k_*(*N*), (2)

*f_k_*^0^ = (*q_k_*(*N* + 1) − *q_k_*(*N* − 1))/2 (3)

Δ*f_k_* = *f_k_*^+^ − *f_k_*^−^ (4)

where *N*—number of electrons in the system, *q_k_*—atom charge (*N* + 1 anion, *N* − 1 cation).

In the present studies, Δ*f_k_* was taken as a convenient parameter for reactivity description, i.e., Δ*f_k_* > 0 indicates the atom in the molecule is more susceptible to nucleophilic attack, Δ*f_k_* < 0 to electrophilic attack, and Δ*f_k_* ≈ 0 indicates a neutral character. The charge distribution was calculated on the M06-2x/aug-cc-pVTZ level of theory in the aqueous phase (C-PCM solvation model) using Charge Model 5 based on Hirshfeld population methodology [56]. The data are presented in Table S1 of the Supplementary Materials (Table S1). In the leading structure (flavan), Δ*f_k_* demonstrated a higher positive value in C6 of the phloroglucinol ring (0.17), and a negative value in C6′ and C3′ of the catechol moiety (−0.19). These carbon atoms appear to be most susceptible to radical action, i.e., *f_k_*^0^ = 0.1, 0.11, and 0.11, respectively.

The extension of the Flv structure to the (−)-epicatechin increased the reactivity of the molecules within the catechol ring. However, the lowest Δ*f_k_* value, i.e., −0.13, was observed for C6′ and C3′, and the highest, i.e., 0.8, to O5′. Moreover, positive *f_k_*^0^ values were noted for all atoms belonging to the catechol moiety, with the highest values assigned to C6′ (0.15) and C3′ (0.14). For the simplest condensed tannin, PCB2, a negative Δ*f_k_* was noted for the catechol C2′ (−0.21) and C5′ (−0.20) atoms of the initial unit (IU) while positive values were recorded for the catechol C6′ (0.13) and O3′ (0.13) atoms of the extended unit (EU^1^). These findings highlight the non-equivalent nucleophilic and electrophilic properties of the procyanidin B2 catechol subunits separated by pyran and phloroglucinol moieties. The prediction of the radical atom susceptibility quantified by *f_k_*^0^ discloses the same atoms as above C2′ (0.11) and C5′ (0.10) of the IU and C6′ (0.7) and O3′ (0.7) of EU^1^. Hence, the IU shows a higher antioxidant ability, i.e., higher radical susceptibility, than the EU. Surprisingly, the addition of another (−)-epicatechin synthon (PCC1) to the PCB2 structure abolishes the reactivity of the IU and shifts it into EU^2^. Again, the lowest Δ*f_k_* values were assigned to C2′ (−0.21) and C5′ (0.20) of the EU^1^ catechol ring; in the case of EU^2^ catechol moiety, the highest Δ*f_k_* was assigned for C6′ (14), C5′ (0.10), O4′ (0.14) and O3′ (0.13). Interestingly, the visible value of *f_k_*^0^ ranged from 0.06 to 0.10 for the same atoms.

For the (−)-epicatechin tetramer, i.e., CTA2, the Δ*f_k_* and *f_k_*^0^ descriptors brought unexpected results. While the nucleophilic and electrophilic parts were close together in PCC1 and PCB2, the EU^1^ and EU^3^ moieties were separated by EU^2^ in CTA2. Hence, for highly condensed tannins, the distribution of reactive moieties is an iterative process, where reactive parts occur after unreactive ones. For CTA2, Δ*f_k_* demonstrated a negative value for C2′ (−0.10) and C5′ (−0.08) of the EU^1^ catechol ring and C5′ (−0.11) of EU^3^ catechol moiety. As expected, the same atoms exhibited the highest *f_k_*^0^ values: 0.05, 0.05, and 0.06, respectively. It should be noted that the electrophilic, nucleophilic, and radical-susceptible properties are widely distributed across the extension units (EU^1^, EU^2^, EU^3^); this predisposes the molecule to stable radical formation and makes it an effective antioxidant.

### 2.5. The Spin Density Distribution of the Flavan and Tannin Derivatives

The Fukui index *f_k_*^0^ is calculated for the equilibrated vertical state. This approach does not account for the non-equilibrated solvent–solute interaction, i.e., the initial moment of radical anion or cation formation, and the unpaired-electron distribution within the molecular structure can change after nuclear relaxation. Therefore, the spin density (*ρS*) was calculated in NE and EQ vertical radical cation/anion states (^NE^VC, ^NE^VA, VC, VA) and after ground state achievement (adiabatic radical cation/anion—AC and AA, respectively). Therefore, the spin distribution was calculated at the M06-2x/aug-cc-pVTZ level of theory in the aqueous phase using the Hirshfeld methodology. Also, due to the system complexity and iterative (−)-epicatechol unit repetition, the *ρS* was simplified and discussed based on the catechol (C), pyran (B), and phloroglucinol (A) rings (Figure 1).

For all discussed forms (^NE^V, VC and AC), the electron loss and radical cation formation distribution of the leading structure, i.e., flavan, is divided in the ratio 25:72 over the pyran and phloroglucinol moiety. However, when the extra electron initially appears in the system (^NE^VA), the spin density is spread over the whole molecule, with subsequent accumulation on the catechol moiety after solvent–solute interaction equilibration and nuclear relaxation. The extension of the Flv structure to (−)-epicatechin occurs through the addition of a hydroxyl group to position C3, C5, C7, and C3′; C4′ disperses the *ρS* over catechol and the phloroglucinol ring in the non-equilibrated vertical radical cation state. The relaxation of solvent and molecular geometry leads to spin density delocalisation on the catechol region. Similarly, a radical anion was found by radical anion analysis (Table 5). However, at ^NE^VA, the unpaired electron was spread over the whole (−)-epicatechin structure.

The smallest of the condensed tannins is procyanidin B2. The loss of the electron causes the spin density to accumulate on the phloroglucinol rings (A^1^ and A^2^) of both the initial unit (0.39) and the extension unit (0.48). As previously, the *ρS* after solvent–solute equilibration (VC) and subsequent radical cation structure relaxation (AC) localised (0.94) on the catechol ring (B2) of EU^1^ (Table 5, Figure 1). Electron loss first causes radical anion formation in the non-equilibrated solvent–solute vertical state, where the spin density is distributed between A^1^(IU) and A^2^(EU^1^) rings. After solvent relaxation, the *ρS* delocalised almost exclusively (0.88) to the C^1^ moiety of PCB2 IU and transferred mainly (0.88) to the catechol ring of the extended unit EU^1^.

The addition of the (−)-epicatechin synthon to the procyanidin B2 moiety results in PCC1 formation. The loss of the electron and the formation of the initial radical cation indicate that spin density is located on three phloroglucinol parts: A^1^ (0.13), A^2^ (0.57), and A^3^ (0.18), which were accumulated after solvent relaxation on catechol ring C^3^ (0.94) of EU^3^ and remain there after PCC1 structure relaxation. Initially, in the ^NE^VA state, the extra electron in the proanthocyanidin C1 was distributed over almost the entire molecule, as shown in Table 5. Solvent relaxation leads to the spin density being concentrated (0.88) on the C^2^ moiety of the EU^2^ of PCC1. Surprisingly, the relaxation of the nucleus to the adiabatic radical anion state transfers the unpaired electron almost completely (0.92) to the catechol ring (C^1^) of the initial unit (IU^1^).

The further addition of another (−)-epicatechin to the trimer leads to CTA2, resulting in electron loss and the stabilisation of spin density on the central part of the cinnamtannin A2, i.e., phloroglucinol moiety A^2^ (0.49) and A^3^ (0.29). This occurs independently of the radical cation states: NE/EQ vertical or adiabatic. Hence, CTA2 appears to be predisposed to radical stabilisation after antioxidative action. However, when an unpaired excess electron appears in the molecular environment initially (^NE^VA), it is then distributed over all subunits (IU, EU^1−3^), as shown in Table 5 and Figure 1. The solvent relaxation (VA) splits the *ρS* to catechol ring C^2^ and C^4^ with a ratio of 0.44:0.46. The subsequent relaxation of the radical anion structure to the ground state forces the spin density to settle in the catechol ring (A^4^) of the terminal extended unit EU^3^.

In conclusion, the comparison of the results above indicates the significant role of the environment in shaping the spin distribution within molecules, thereby altering their chemical properties, as shown in Table 5.

It should be pointed out that the present theoretical studies did not address the biological aspects of the compounds, i.e., their bioavailability, metabolism, pharmacokinetics, membrane transport, intracellular accumulation, microbiome transformation, and toxicity. However, the previous studies demonstrated that highly polymerised procyanidins exhibit limited intestinal absorption and extensive microbiome-dependent metabolism [13,46,47]. Furthermore, flavonoids and tannins may exhibit both antioxidant and pro-oxidant behaviour depending on concentration, transition metal availability, and cellular redox status [11,20,21]. Moreover, the relationship between antioxidant properties and radioprotective or chemotherapeutic effects is highly complex and depends on multiple biological variables, including ROS signalling, mitochondrial activity, apoptosis regulation, and tumour-specific metabolism [9,57,58]. Moreover, previous experimental studies have found PCC1 and related flavonoids to exhibit senotherapeutic or apoptosis-related activity [15]. Therefore, in this context, the present theoretical study should not be considered directly as evidence of therapeutic efficacy, but as indicative of the properties of the tested compounds for use in potential therapeutic strategies.

## 3. Discussion

Each cell in the human body is continuously exposed to various physical and chemical harmful factors [57]. Therefore, cells have developed various protective mechanisms based on small molecules, enzymes, and DNA repair systems [59]. While all molecules act in concert, enzymes are unable to quench short-lived radicals, such as hydroxyl radicals (HO•) [60]. As such, the low-molecular-weight molecules, i.e., antioxidants, are on the front line on both sides of the cellular membrane.

Antioxidants play a crucial role in maintaining “radical” homeostasis. One particularly common group of antioxidants consumed with food are the procyanidins, found in tea, cocoa, grapes, and apple seeds, which belong to the condensed tannins, e.g., (−)-epicatechin. Procyanidins are believed to exert their free radical scavenging by hydrogen atom transfer (HAT), sequential electron transfer proton transfer (SET-PT) and sequential proton-loss electron-transfer (SPL-ET) [25,26]. Previous theoretical studies indicate that their protective role against ROS, for example, increases with the degree of polymerisation as follows: monomer < dimer < trimer [19]. It should be noted that cells are also susceptible to damage from physical factors, such as ionising radiation or UV. Interest has therefore grown in identifying new methods of protecting the native extra- and intracellular matrix, especially during radiotherapy. One particularly promising approach concerns the use of safe, naturally derived molecules, such as procyanidins [61].

The HAT, SET-PT, and SPLET mechanisms are commonly used to describe the antioxidant activity of phenolic compounds and flavonoids. However, a full mechanistic comparison requires additional thermodynamic parameters such as bond dissociation enthalpy (BDE) for HAT, proton dissociation enthalpy (PDE) for SET-PT, and proton affinity (PA) for SPLET. These descriptors were not calculated in the present study: our findings should be interpreted primarily in terms of radical stabilisation and electron accepting/donating capacity. The above is consistent with previous theoretical studies showing that ionisation potential and electron affinity are directly related to charge transfer antioxidant pathways [26,62]. Also, DFT studies on procyanidins indicate that BDE serves as the key descriptor for HAT, PA for the first step of SPLET, and IP for SET-related mechanisms [16,19].

The ability of the discussed molecules to quench the unwanted products of water radiolysis and prevent self-propagating free radical generation can be estimated by ionisation potential and electron affinity. This is addressed in the present study by theoretical investigations at the M06-2x/aug-cc-pVTZ level of theory in the aqueous phase. The calculated electronic properties indicate that the condensed tannins PCB2, PCC1, and CTA2 are characterised by lower vertical and adiabatic ionisation potentials than their monomers and are therefore better radical scavengers (Table 3). Moreover, the calculated ^NE^VIP, ^EQ^VIP, and AIP values were found to be lower than the assigned values for guanine (Gua), indicating that they may perform a genome-protecting role. Gua has the lowest IP among the canonical nucleobases. On the other hand, the highest electron affinity and the predisposition to the solvated electron quenching were calculated for (−)-epicatechin (1.18 eV) and its trimer, PCC1 (1.15 eV). However, the highest non-equilibrated vertical electron affinity was demonstrated by cinnamtannin A2, i.e., 0.12 eV. These findings indicate that the reactivity of procyanidins depends on their structural complexity.

The condensed tannin subunits do not exhibit equal reactivity. The IU of dimers (PCB2) showed higher reactivity, while for PCC1 it was extended to the intermediate (−)-epicatechin moiety, i.e., EU^1^. The findings presented here, indicate the following conformational scheme between subunits for the neutral/cation/anion forms, respectively: PCB2 compact in all cases; PCC1 extended–compact/extended–extended/compact–compact; CTA2 solely extended–compact–extended. For all the discussed structures, no imaginary frequencies were found, indicating the ground-state geometry. However, in previous studies, Mendoza-Wilson et al. assigned the compact and compact–compact conformations for the non-charged dimer and trimer at the M05-2x/6-31+G** level in the aqueous phase [19].

**Figure 3:**
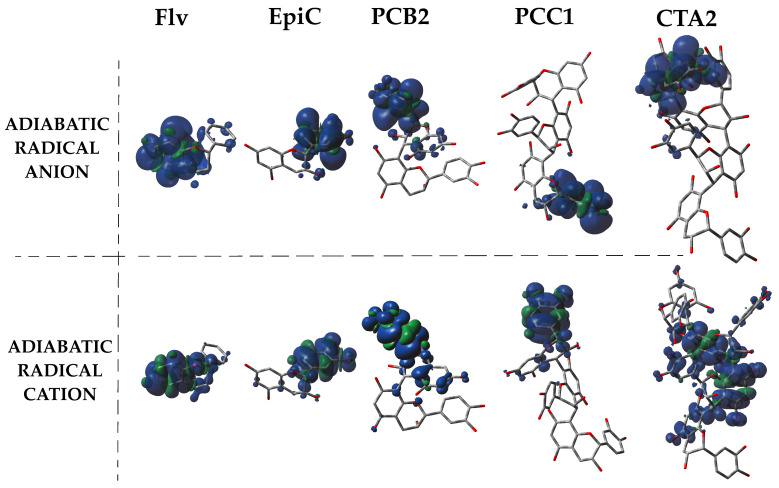
The graphical spin density distribution of the adiabatic radical anion and cation calculated at the M06-2x/aug-cc-pVTZ level of theory in the aqueous phase.

These results were well supported by the global reactivity descriptor calculations. The scavenging (quenching) of free radicals, or the termination of their propagation by the discussed antioxidants, is correlated with the electronic chemical potential (μ), i.e., the tendency of a molecule to lose or adopt an electron. The obtained results (Table 4) indicate that all (−)-epicatechin derivatives possess high negative μ values, suggesting their good electron acceptor properties. Among the selected antioxidants, hardness was found to decrease as softness increased, accompanied by structure relaxation (vertical → adiabatic state). However, similar to other small-molecular-weight antioxidants, (−)-epicatechin and condensed tannins have higher hardness and lower softness [50]. Their presence in the extracellular and intracellular matrices can protect the cell membrane (bilipid layer) and cell environment against free radicals, such as ROS and RNS. The spin density analysis found them to have various degrees of condensation (Figure 3 and Table 5), which can predict their different roles in biological systems. It should be pointed out that as flavonoids and tannins have large numbers of phenolic groups, they are able to coordinate transition metal ions, e.g., Fe^2+,3+^, Cu^2+^, Mn^2+^; such coordination can prevent free radical generation occurring via the Haber–Weiss reaction [61].

It should be noted that condensed tannins, such as procyanidins (PCB2, PCC1, and CTA2), are absorbed from the small intestine at only 10% of the level of (−)-epicatechin, and that procyanidins with polymerisation levels higher than tetramers are not absorbed [63]. The above corresponds well with their calculated molecular volume and length (21.15 Å), which is close to the pore inner diameter (26 Å) for CTA2. In addition, the discussed tannins did not appear to be digested to monomeric forms by the microbiome, nor influence procyanidin levels in the blood or ECM. However, the intake of these antioxidants with food can modulate the gut microbiota composition [64]. Therefore, PCC1 demonstrates the lowest AIP and highest AEA among all discussed condensed tannins, and its role in free radical cell homeostasis and ECM stability is of considerable interest. Moreover, previous studies have shown that PCC1 possesses important senotherapeutic activity. Xu et al. report the selective elimination of senescent cells by procyanidin C1 and attribute this to ROS accumulation, mitochondrial dysfunction, cytochrome C release, and the activation of pro-apoptotic pathways involving PUMA and NOXA [15]. Additionally, procyanidins and other flavonoids have been found to modulate the Bcl-2/Bax balance, caspase activation, NF-κB suppression, and depolarisation of the mitochondrial bi-layer membrane [14,17,18]. Furthermore, flavonoids demonstrate concentration-dependent antioxidant and pro-oxidant activity, especially in environments enriched with transition metal ions [11,20,21]. These biological observations may, to an extent, be explained by our present findings, particularly those regarding the electronic properties of the compounds. PCC1 demonstrated the lowest adiabatic ionisation potential (AIP = 6.08 eV), while CTA2 discloses the smallest HOMO–LUMO energy gap (Δ*E^H-L^* = 6.77 eV). Such parameters indicate enhanced electron-transfer ability, increased chemical reactivity, and the stabilisation of radical intermediates. These parameters may influence the modulation of ROS/RNS homeostasis and redox-sensitive apoptotic signalling pathways in the extracellular and intracellular environments. Additionally, within condensed tannins, PCC1 has been found to have one of the highest adiabatic electron affinities (AEA = 1.15 eV), indicating its extra electron adoption ability. This may be significant under an oxidative stress environment, as senescent cells typically display elevated basal ROS levels and dysfunctional mitochondrial metabolism. Compounds with high electron affinity and efficient spin-density delocalisation may therefore interfere with the redox equilibrium selectively in senescent or cancer cells, promoting apoptosis while simultaneously protecting normal cells against uncontrolled oxidative damage to DNA, lipids, and proteins. The spin-density distribution over catechol and phloroglucinol moieties discussed herein, together with their high electrophilicity ω ≈ 2.3–2.7 eV, confirms that condensed tannins are able to stabilise radical states and participate in environmental electron-transfer processes. Similar relationships between electronic structure, antioxidant/pro-oxidant balance and biological activity have been noted for flavonoids and other polyphenols [16,19,25,26,50]. Moreover, some polyphenolic compounds are capable of undergoing efficient electron transfer and radical stabilisation; these have been proposed as modulators of the mitochondrial oxidative stress, DNA protection, and apoptosis induction pathways associated with ageing and carcinogenesis [9,48,57]. Due to the fact that oxidative stress and redox imbalance are triggers of senescence-associated signalling and senescence-associated secretory phenotype, i.e., SASP-related pathways [65,66,67,68], the electronic features discussed herein may offer an explanation for the senomorphic and senotherapeutic properties observed for condensed tannins.

## 4. Materials and Methods

The initial geometries of: Flavan, (−)-epicatechin, procyanidin B2, procyanidin C1, and cinnamtannin A2 were constructed using GaussView 5 software [69]. All structures are shown in Figure 1.

All geometry and energy calculations were performed using the Global Hybrid Functional (Minnesota M06-2x with 54% of Hartree-Fock exchange). The mean unsigned errors and balanced mean unsigned errors were estimated in benchmarks for M06-2x functional on the level of 1–3 kcal·mol^−1^, which yields high accuracy for the main group [29]. Due to time-consumption (cost) reduction, the 6-31++G** Pople’s split-valence double-zeta basis set was used for geometry optimisation in the aqueous phase [70,71]. The solvent was described using the conductor-like continuum polarisable continuum model (C-PCM), developed by Tomasi, with a water dielectric constant ε = 78.4 [72]. To confirm the ground state of the obtained geometries, the Hessian matrix was calculated using the same functional and basis set in the aqueous phase given above. For all obtained structures, no imaginary frequency was found. All energies have been calculated at the M06-2x/aug-cc-pVTZ level of theory (using the following basis set for heavy atoms and hydrogen: *5s4p3d2f* and *4s3p2d*, respectively) in the aqueous phase [73]. It should be pointed out that all geometry optimisations, frequency calculations, and single-point energy calculations were performed using the standard M06-2X functional, together with the explicitly specified 6-31++G** and aug-cc-pVTZ basis sets, without any custom basis reduction or modifications.

For all the ground state geometries of neutral and radical cation and anions, as well as the corresponding vertical states, a charge and spin distribution analysis was performed. This was based on an extension of the Hirshfeld population analysis Charge Model 5 at the M06-2x/aug-cc-pVTZ level of theory in the aqueous phase [56].

The electronic properties of the selected antioxidants, i.e., their vertical and adiabatic electron affinities and ionisation potentials, were calculated at the M06-2x/aug-cc-pVTZ level of theory in the aqueous phase. The solvent effect was analysed in non-equilibrium (NE) and equilibrated (EQ) solvent–solute interaction modes using the conductor-like polarisable continuum model, as described previously [74,75]. The energy of the molecule in the non-equilibrated mode was calculated using the save-read procedures.

To simplify the procedure, the following energy notation was used: *E_Geometry_^Charge^*. In this notation, *E*_0_^0^ corresponds to a neutral molecule in its ground state; *E*_0_^+^*/E*_0_^−^ are related to the vertical cation/anion in the equilibrated solvent–solute interaction mode; *^NE^E*_0_^+^ and *^NE^E*_0_^−^ represent the vertical cation/anion in the non-equilibrated (NE) solvent–solute interaction mode. Finally, E_+_^+^ and E_−_^−^, represent the energy of the ground state geometry of the radical cation and anion (adiabatic state), respectively [50].

Therefore, the (a) vertical ionisation potential in the NE state (VIP^NE^) is the energy difference between *E*_0_^0^ and *^NE^E*_0_^+^; (b) the vertical ionisation potential in the EQ state, VIP^EQ^ = *E*_0_^0^ − *E*_0_^+^; (c) the vertical electron affinity in the NE state, VEA^NE^ = ^NE^E_0_^−^ − E_0_^0^; (d) the vertical electron affinity in the EQ state, VEA = *E*_0_^−^ − *E*_0_^0^; (e) the adiabatic ionisation potential, AIP = *E*_+_^+^ − *E*_0_^0^; and (f) the adiabatic electron affinity, AEA = *E*_0_^0^ − *E*_−_^−^.

For this study, the energies of antioxidants and all electronic properties are given in [eV]. All the discussed theoretical calculations were performed using the Gaussian G16 (version C.01) software package [76].

## Figures and Tables

**Table 1:** Molecular Volume (Å^3^/mol) calculated (tight mode) on M06-2x/6-31++G** level of theory in aqueous phase. Molecule length (Å), and Root Mean Square Deviation (RMSD) of all atomic positions in (Å^2^), calculated for neutral and radical anionic/cationic forms of Flavan (Flv), (−)-epicatechin (EpiC), procyanidin B2 (PCB2), procyanidin C1 (PCC1), and cinnamtannin A2 (CTA2) calculated on M06-2x/6-31++G** level of theory in aqueous phase.

Name	Molecular Volume (Å^3^/mol)			Length of Molecule (Å)			RMSD(Å^2^)	
	Neutral	Anion	Cation	Neutral	Anion	Cation	Anion v. Neutral	Cation v. Neutral
Flv	939.74	1054.65	877.85	9.47	9.35	9.72	0.2394	0.2983
EpiC	1043.66	1159.18	1190.14	11.22	11.18	10.89	0.1888	0.2758
PCB2	2208.15	2217.77	2231.21	15.00	14.47	14.16	0.4113	0.4457
PCC1	3307.29	3296.71	3489.17	18.57	17.60	18.55	3.8027	0.3514
CTA2	4268.33	4367.586	4252.64	21.15	21.13	20.65	0.9160	0.3740

**Table 2:** The selected geometrical parameters, i.e., interflavonoid bond δ (C4–C8) in the [Å] and dihedral angle χ (C3–C4–C8–C9) in the aqueous phase of procyanidin B2, C1, and cinnamtannin A2 in their neutral (N), anion (A), and cation (C) radical forms. Data calculated based on M06-2x/6-31++G** level of theory.

Name	Interflavonoid Bond (Å)			Interflavonoid Dihedral Angle (°)		
	δ^1^	δ^2^	δ^3^	χ^1^	χ^2^	χ^3^
PCB2	1.52**^N^**/1.52**^A^**/1.52**^C^**			88.4**^N^**/81.46**^A^**/89.3**^C^**		
PCC1	1.52/1.52/1.52	1.52/1.52/1.52		−89.8/−87.8/−88.4	79.7/−92.0/80.9	
CTA2	1.52/1.52/1.52	1.52/1.52/1.50	1.52/1.51/1.51	−88.8/−88.8/−89.9	68.3/71.4/67.7	−117.0/−118.5/−112.6

**Table 3:** The electronic properties, in [eV], of flavan, (−)-epicatechin, procyanidin B2, procyanidin C1, and cinnamtannin A2, i.e., the vertical/adiabatic ionisation potential (VIP/AIP) and the vertical/adiabatic electron affinities (VEA/AEA), as well as energies of highest occupied and lowest unoccupied molecular orbitals (HOMO, LUMO) together with their energetic gap Δ*E^H-L^* in [eV], calculated at the M06-2x/aug-cc-pVTZ level of theory in the aqueous phase.

Compound	HOMO	LUMO	Δ*E^H-L^*	VIP^NE^	VIP^EQ^	AIP	VEA^NE^	VEA^EQ^	AEA
Flv	−7.65	−0.18	7.47	7.53	6.48	6.23	−0.04	0.93	1.05
EpiC	−7.50	−0.26	7.24	7.49	6.44	6.15	−0.01	0.88	1.18
PCB2	−7.29	−0.33	6.96	7.21	6.39	6.10	0.02	0.80	0.63
PCC1	−7.24	−0.38	6.86	7.12	6.36	6.08	0.09	0.82	1.15
CTA2	−7.17	−0.40	6.77	7.05	6.35	6.11	0.12	0.81	0.84
Gua [38]	−8.31	−0.77	7.53	8.03	6.95	6.42	−0.09	0.31	0.92

**Table 4:** The reactivity descriptors, in eV, of flavan, (−)-epicatechin, procyanidin B2, procyanidin C1, and cinnamtannin A2 calculated at the M06-2x/aug-cc-pVTZ level of theory in the aqueous phase. The valence molecular orbital energy has been used according to Koopmans’ theorem (Koopmans’ Th.). The energy differences between vertical ionisation potential and vertical electron affinity were calculated in non-equilibrated/equilibrated solvent–solute interactions, denoted as, vertical NE and EQ, respectively. The reactivity descriptors are also calculated after radical anion/cation geometry relaxation to the ground state and noted as adiabatic.

Comp.	Model/State	Descriptors [eV]				Comp.	Model/State	Descriptors [eV]			
		*η*	*S*	*μ*	*ω*			*η*	*S*	*μ*	*ω*
Flv	Koopmans’.Th	3.74	0.27	−3.92	2.05	PCC1	Koopmans.Th	3.43	0.29	−3.81	2.12
	Vertical NE	3.79	0.26	−3.75	1.85		Vertical NE	3.52	0.28	−3.61	1.85
	Vertical EQ	2.78	0.36	−3.71	2.47		Vertical EQ	2.77	0.36	−3.59	2.33
	Adiabatic	2.59	0.39	−3.64	2.56		Adiabatic	2.47	0.41	−3.62	2.65
EpiC	Koopmans’.Th	3.62	0.28	−3.88	2.08	CTA2	Koopmans’.Th	3.39	0.30	−3.79	2.12
	Vertical NE	3.75	0.27	−3.74	1.87		Vertical NE	3.47	0.29	−3.59	1.85
	Vertical EQ	2.78	0.36	−3.66	2.41		Vertical EQ	2.77	0.36	−3.58	2.31
	Adiabatic	2.49	0.40	−3.67	2.70		Adiabatic	2.64	0.38	−3.48	2.29
PCB2	Koopmans’.Th	3.48	0.29	−3.81	2.09						
	Vertical NE	3.61	0.28	−3.61	1.80						
	Vertical EQ	2.80	0.36	−3.60	2.31						
	Adiabatic	2.74	0.37	−3.37	2.07						

**Table 5:** The Hirshfeld spin distribution (summed to the pyran, catechol, and phloroglucinol ring) of selected antioxidants calculated at the M06-2x/aug-cc-pVTZ level of theory in the aqueous phase and given [au]. VC—vertical cation, VA—vertical anion, AC—adiabatic cation, AA—adiabatic anion, NE—non-equilibrated, EQ—equilibrated solvent–solute interaction mode.

Flavan							(−)-Epicatechin						
Ring (Unit)	^NE^VC	VC	AC	^NE^VA	VA	AA	Ring (Unit)	^NE^VC	VC	AC	^NE^VA	VA	AA
Pyran	0.27	0.25	0.23	0.24	0.08	0.08	Pyran	0.11	0.04	0.03	0.22	0.08	0.10
Catechol	0.04	0.03	0.02	0.42	0.88	0.09	Catechol	0.46	0.95	0.96	0.46	0.91	0.90
Phloroglucinol	0.70	0.72	0.75	0.34	0.04	0.83	Phloroglucinol	0.42	0.01	0.01	0.32	0.01	0.01
Procyanidin B2							Cinnamtannin A2						
Ring (Unit)	^NE^VC	VC	AC	^NE^VA	VA	AA	Ring (Unit)	^NE^VC	VC	AC	^NE^VA	VA	AA
Pyran (IU)	0.02	0.00	0.00	0.04	0.06	0.00	Pyran (IU)	0.00	0.00	0.00	0.03	0.00	0.00
Catechol (IU)	0.01	0.00	0.00	0.34	0.88	0.00	Catechol (IU)	0.01	0.00	0.00	0.05	0.00	0.00
Phloroglucinol (IU)	0.39	0.01	0.00	0.03	0.01	0.01	Phloroglucinol (IU)	0.08	0.07	0.01	0.09	0.00	0.00
Pyran (EU^1^)	0.10	0.04	0.03	0.06	0.00	0.07	Pyran (EU^1^)	0.01	0.01	0.01	0.03	0.02	0.00
Catechol (EU^1^)	0.01	0.90	0.94	0.41	0.01	0.88	Catechol (EU^1^)	0.02	0.03	0.00	0.06	0.44	0.03
Phloroglucinol (EU^1^)	0.48	0.04	0.03	0.11	0.04	0.04	Phloroglucinol (EU^1^)	0.49	0.50	0.47	0.10	0.00	0.00
Procyanidin C1							Pyran (EU^2^)	0.05	0.04	0.05	0.05	0.00	0.01
Ring (Unit)	^NE^VC	VC	AC	^NE^VA	VA	AA	Catechol (EU^2^)	0.01	0.02	0.01	0.21	0.00	0.04
Pyran (IU)	0.01	0.00	0.00	0.06	0.00	0.07	Phloroglucinol (EU^2^)	0.29	0.28	0.44	0.13	0.00	0.00
Catechol (IU)	0.01	0.00	0.00	0.10	0.00	0.92	Pyran (EU^3^)	0.01	0.01	0.01	0.02	0.06	0.05
Phloroglucinol (IU)	0.13	0.00	0.00	0.19	0.01	0.01	Catechol (EU^3^)	0.00	0.00	0.00	0.12	0.46	0.85
Pyran (EU^1^)	0.02	0.00	0.00	0.05	0.05	0.00	Phloroglucinol (EU^3^)	0.02	0.02	0.01	0.11	0.01	0.01
Catechol (EU^1^)	0.02	0.00	0.00	0.14	0.88	0.00							
Phloroglucinol (EU^1^)	0.57	0.00	0.00	0.14	0.01	0.00							
Pyran (EU^2^)	0.06	0.04	0.03	0.03	0.00	0.00							
Catechol (EU^2^)	0.01	0.93	0.94	0.08	0.00	0.00							
Phloroglucinol (EU^2^)	0.18	0.02	0.03	0.21	0.04	0.00							

## Data Availability

Data are contained within the article and Supplementary Materials.

## Supplementary Materials

The following supporting information can be downloaded at: https://www.mdpi.com/article/10.3390/molecules31111876/s1, Table S1: The Fukui reactivity index provides information about the reactivity of atoms in the molecule. The atom radical (*f*_k_^0^) attacks susceptibility factors and dual descriptors (Δ*f_k_*), and were calculated thus: *f_k_*^+^ = *q_k_*(*N* − 1) − *q_k_*(*N*), *f_k_*^−^ = *q_k_*(*N* + 1) − *q_k_*(*N*), *f_k_*^0^ = (*q_k_*(*N* + 1) − *q_k_*(*N* − 1))/2 Δ*f_k_* = *f_k_*^+^ − *f_k_*^−^ where *N*—number of electrons in the system, *q_k_*—atom charge (*N* + 1 anion, *N* − 1 cation). The charge distribution was calculated on the M06-2x/aug-cc-pVTZ level of theory in the aqueous phase using Charge Model 5 based on the Hirshfeld population methodology; Table S2: The Electronic state Energy in Hartree and Dipole Moment (DM) in Debye was calculated on the M06-2x/aug-cc-pVTZ level of theory in the aqueous phase using the non-equilibrated and equilibrated solvent–solute interaction. EpiC: (−)-epicatechin; PCB2: pprocyanidin B2; PCC1: Procyanidin C1; CTA2: cinnamtannin A2. Pdb structures of all discussed molecules (mentioned above) in their anion (A), cation (C), and neutral (N) forms.
