# Supplementary material for: A Theoretical Prediction of the Antioxidant and Electronic Properties of Epicatechin, Procyanidin B2, Procyanidin, C1 and Cinnamtannin A2
Source: Molecules. 2026 May 29;31(11):1876. doi: 10.3390/molecules31111876 (PMC13258072; doi:10.3390/molecules31111876)
Supplement: Supplementary file 1 [file molecules-31-01876-s001.zip › molecules-4302581-supplementary.pdf]

Supplementary Materials

A theoretical prediction of the antioxidant and electronic properties of Epicatechin, Procyanidin B2, Procyanidin C1 and Cinnamtannin A2

Boleslaw T. Karwowski

Table S1

The Fukui reactivity index, provides information about the reactivity of atoms in the molecule. The atom radical (f_k^0) attack susceptibility factors and dual descriptor (Δf_k), were calculated thus:

$f_k^+ = q_k(N\text{-}1) - q_k(N)$, $f_k^- = q_k(N\text{+}1) - q_k(N)$, $f_k^0 = (q_k(N\text{+}1) - q_k(N\text{-}1))/2$ $\Delta f_k = f_k^+ - f_k^-$

N - number of electrons in the system, q_k - atom charge (N+1 anion, N-1 cation).

The charge distribution calculated on M06-2x/aug-cc-pVTZ level of theory in aqueous phase using Charge Model 5 based on Hirshfeld population methodology.

**Flavan**						
**Ring**	**Atom Number**	**Neutral**	**Vertical Cation**	**Vertical Anion**	Δf_k	f_k^0
**Phloroglucinol (A^1)**	**C5**	0.00	0.08	-0.01	0.07	0.05
	**C6**	-0.02	0.17	-0.04	0.17	0.10
	**C7**	0.00	0.09	-0.02	0.08	0.06
	**C8**	-0.01	0.10	-0.03	0.10	0.07
	**C9**	0.08	0.19	0.08	0.11	0.05
	**C10**	-0.03	0.04	-0.04	0.07	0.04
**Pyran (B^1)**	**O1**	-0.23	-0.12	-0.24	0.10	0.06
	**C2**	0.14	0.19	0.10	0.00	0.04
	**C3**	0.04	0.09	0.01	0.01	0.04
	**C4**	0.04	0.10	0.01	0.04	0.04
**Catechol (C^1)**	**C1’**	-0.02	-0.02	-0.08	-0.06	0.03
	**C2’**	-0.01	0.01	-0.10	-0.08	0.05
	**C3’**	0.01	0.02	-0.19	-0.19	0.11
	**C4’**	0.00	0.02	-0.20	-0.12	0.08
	**C5’**	0.00	0.02	-0.14	-0.10	0.07
	**C6’**	0.00	0.02	-0.11	-0.19	0.11

(-)-Epicatechin						
**Ring**	**Atom Number**	**Neutral**	**Vertical Cation**	**Vertical Anion**	Δf_k	f_k^0
**Phloroglucinol (A[1])**	**C5**	0.09	0.09	0.08	0.00	0.00
	**O5**	-0.03	-0.02	-0.03	0.00	0.01
	**C6**	-0.04	-0.03	-0.05	0.00	0.01
	**C7**	0.08	0.08	0.08	0.00	0.00
	**O7**	-0.05	-0.04	-0.05	0.00	0.01
	**C8**	-0.04	-0.03	-0.05	0.00	0.01
	**C9**	0.09	0.09	0.09	0.00	0.00
	**C10**	-0.06	-0.06	-0.06	0.00	0.00
**Pyran (B[1])**	**O1**	-0.13	-0.11	-0.17	-0.02	0.03
	**C2**	0.14	0.17	0.10	-0.01	0.04
	**C3**	0.12	0.14	0.10	0.00	0.02
	**O3**	-0.22	-0.21	-0.23	0.00	0.01
	**C4**	0.03	0.05	0.01	0.00	0.02
**Catechol (C[1])**	**C1’**	-0.03	0.05	-0.09	0.03	0.07
	**C2’**	-0.01	0.13	-0.10	0.05	0.11
	**C3’**	0.01	0.09	-0.21	-0.13	0.15
	**C4’**	0.07	0.18	-0.01	0.03	0.09
	**O4’**	-0.02	0.11	-0.09	0.06	0.10
	**C5’**	0.07	0.18	0.00	0.03	0.09
	**O5’**	-0.06	0.08	-0.12	0.08	0.10
	**C6’**	0.00	0.07	-0.20	-0.13	0.14

Procyanidin B2 (PCB2)						
**Ring**	**Atom Number**	**Neutral**	**Vertical Cation**	**Vertical Anion**	Δf_k	f_k^0
**Phloroglucinol (A[1])**	**C5**	-0.20	-0.20	-0.20	0.00	0.00
	**O5**	0.14	0.14	0.10	-0.03	0.02
	**C6**	0.12	0.12	0.10	-0.02	0.01
	**C7**	-0.14	-0.14	-0.16	-0.02	0.01
	**O7**	0.04	0.04	0.03	-0.01	0.01
	**C8**	0.08	0.09	0.08	0.00	0.00
	**C9**	-0.03	-0.03	-0.04	0.00	0.00
	**C10**	-0.03	-0.03	-0.04	0.00	0.01
**Pyran (B[1])**	**O1**	0.08	0.08	0.08	0.00	0.00
	**C2**	-0.01	-0.01	-0.02	0.00	0.00
	**C3**	-0.05	-0.05	-0.05	0.00	0.00
	**O3**	0.09	0.09	0.09	0.00	0.00
	**C4**	-0.06	-0.06	-0.06	0.00	0.00
**Catechol (C[1])**	**C1’**	-0.03	-0.03	-0.08	-0.05	0.03
	**C2’**	-0.01	-0.01	-0.09	-0.08	0.04
	**C3’**	0.00	0.00	-0.20	-0.20	0.10
	**C4’**	0.06	0.06	-0.01	-0.07	0.04
	**O4’**	-0.03	-0.03	-0.10	-0.07	0.03
	**C5’**	0.07	0.07	-0.01	-0.07	0.04
	**O5’**	-0.06	-0.06	-0.12	-0.06	0.03
	**C6’**	0.00	0.00	-0.21	-0.21	0.11
**Phloroglucinol (A[2])**	**C5**	0.09	0.10	0.09	0.00	0.00
	**O5’**	-0.02	-0.01	-0.03	0.00	0.01
	**C6**	-0.04	-0.02	-0.05	0.01	0.01
	**C7**	0.08	0.09	0.08	0.00	0.01
	**O7’**	-0.05	-0.04	-0.06	0.00	0.01
	**C8**	-0.03	-0.02	-0.05	0.00	0.01
	**C9**	0.09	0.09	0.09	0.00	0.00
	**C10**	-0.05	-0.04	-0.05	0.00	0.00
**Pyran (B[2])**	**O1**	-0.23	-0.21	-0.23	0.01	0.01
	**C2**	0.14	0.17	0.14	0.03	0.02
	**C3**	0.12	0.13	0.11	0.01	0.01
	**O3**	-0.14	-0.13	-0.14	0.01	0.01
	**C4**	0.02	0.03	0.02	0.00	0.00
**Catechol (C[2])**	**C1’**	-0.03	0.05	-0.03	0.08	0.04
	**C2’**	0.00	0.06	-0.01	0.06	0.03
	**C3’**	-0.02	0.11	-0.02	0.13	0.07
	**C4’**	0.07	0.17	0.07	0.10	0.05
	**O4’**	0.00	0.08	0.00	0.08	0.04
	**C5’**	0.06	0.17	0.06	0.11	0.05
	**O5’**	-0.06	0.07	-0.06	0.13	0.07
	**C6’**	-0.03	0.10	-0.03	0.12	0.06

Procyanidin C1 (PCC1)						
**Ring**	**Atom Number**	**Neutral**	**Vertical Cation**	**Vertical Anion**	***Δf_k***	***f_k⁰***
**Phloroglucinol (A[1])**	**C5**	0.08	0.08	0.08	0.00	0.00
	**O5**	-0.03	-0.03	-0.03	0.00	0.00
	**C6**	-0.04	-0.04	-0.04	0.00	0.00
	**C7**	0.08	0.08	0.08	0.00	0.00
	**O7**	-0.02	-0.02	-0.02	0.00	0.00
	**C8**	-0.05	-0.05	-0.05	0.00	0.00
	**C9**	0.09	0.09	0.09	0.00	0.00
	**C10**	-0.06	-0.06	-0.06	0.00	0.00
**Pyran (B[1])**	**O1**	-0.21	-0.21	-0.21	0.00	0.00
	**C2**	0.15	0.15	0.14	0.00	0.00
	**C3**	0.12	0.12	0.12	0.00	0.00
	**O3**	-0.09	-0.09	-0.09	0.00	0.00
	**C4**	0.03	0.03	0.03	0.00	0.00
**Catechol (C[1])**	**C1’**	-0.03	-0.03	-0.03	0.00	0.00
	**C2’**	-0.02	-0.02	-0.02	0.00	0.00
	**C3’**	0.07	0.07	0.07	0.00	0.00
	**C4’**	-0.04	-0.04	-0.04	0.00	0.00
	**O4’**	0.06	0.06	0.06	0.00	0.00
	**C5’**	-0.03	-0.03	-0.03	0.00	0.00
	**O5’**	0.00	0.00	0.00	0.00	0.00
	**C6’**	-0.02	-0.01	-0.02	0.00	0.00
**Phloroglucinol (A[2])**	**C5**	0.08	0.09	0.08	0.00	0.00
	**O5’**	-0.04	-0.03	-0.04	0.00	0.00
	**C6**	-0.04	-0.03	-0.05	0.00	0.01
	**C7**	0.08	0.08	0.07	0.00	0.00
	**O7’**	-0.02	-0.02	-0.03	0.00	0.00
	**C8**	-0.05	-0.05	-0.05	0.00	0.00
	**C9**	0.09	0.09	0.09	0.00	0.00
	**C10**	-0.05	-0.05	-0.06	0.00	0.00
**Pyran (B[2])**	**O1**	-0.20	-0.20	-0.20	0.00	0.00
	**C2**	0.13	0.13	0.10	-0.03	0.02
	**C3**	0.11	0.11	0.09	-0.02	0.01
	**O3**	-0.13	-0.13	-0.15	-0.02	0.01
	**C4**	0.02	0.02	0.02	0.00	0.00
**Catechol (C[2])**	**C1’**	-0.03	-0.03	-0.09	-0.05	0.03
	**C2’**	-0.02	-0.02	-0.10	-0.08	0.04
	**C3’**	0.00	0.00	-0.20	-0.20	0.10
	**C4’**	0.06	0.06	-0.01	-0.07	0.04
	**O4’**	-0.03	-0.03	-0.09	-0.06	0.03
	**C5’**	0.07	0.07	0.00	-0.07	0.04
	**O5’**	-0.06	-0.06	-0.12	-0.06	0.03
	**C6’**	0.00	0.00	-0.21	-0.21	0.10
**Phloroglucinol (A[3])**	**C5**	0.09	0.09	0.09	0.00	0.00
	**O5**	-0.02	-0.02	-0.03	0.00	0.01
	**C6**	-0.04	-0.02	-0.05	0.01	0.01
	**C7**	0.08	0.08	0.07	0.00	0.01

	**O7**	-0.05	-0.04	-0.05	0.00	0.01
	**C8**	-0.04	-0.03	-0.06	-0.01	0.02
	**C9**	0.09	0.09	0.09	0.00	0.00
	**C10**	-0.05	-0.05	-0.05	0.00	0.00
**Pyran (B^3)**	**O1**	-0.22	-0.21	-0.22	0.01	0.00
	**C2**	0.14	0.17	0.14	0.03	0.02
	**C3**	0.12	0.13	0.11	0.01	0.01
	**O3**	-0.13	-0.12	-0.14	0.01	0.01
	**C4**	0.02	0.03	0.02	0.00	0.00
**Catechol (C^3)**	**C1’**	-0.03	0.05	-0.03	0.08	0.04
	**C2’**	-0.02	0.12	-0.02	0.14	0.07
	**C3’**	0.00	0.08	0.00	0.08	0.04
	**C4’**	0.06	0.17	0.06	0.11	0.05
	**O4’**	-0.03	0.10	-0.03	0.13	0.06
	**C5’**	0.07	0.17	0.07	0.10	0.05
	**O5’**	-0.06	0.07	-0.06	0.14	0.07
	**C6’**	0.00	0.07	-0.01	0.07	0.04

Cinnamtannin (CTA2)						
**Ring**	**Atom Number**	**Neutral**	**Vertical Cation**	**Vertical Anion**	***Δf_k***	***f_k⁰***
**Phloroglucinol (A¹)**	**C5**	0.08	0.09	0.08	0.01	0.01
	**O5**	-0.03	-0.02	-0.03	0.01	0.01
	**C6**	-0.04	-0.02	-0.04	0.01	0.01
	**C7**	0.08	0.09	0.08	0.01	0.00
	**O7**	-0.02	-0.01	-0.02	0.01	0.01
	**C8**	-0.05	-0.04	-0.05	0.01	0.00
	**C9**	0.09	0.10	0.09	0.00	0.00
	**C10**	-0.06	-0.05	-0.06	0.01	0.00
**Pyran (B¹)**	**O1**	-0.21	-0.21	-0.21	0.01	0.00
	**C2**	0.15	0.15	0.14	0.00	0.00
	**C3**	0.12	0.12	0.12	0.00	0.00
	**O3**	-0.09	-0.09	-0.09	0.00	0.00
	**C4**	0.03	0.04	0.03	0.01	0.00
**Catechol (C¹)**	**C1’**	-0.03	-0.03	-0.03	0.00	0.00
	**C2’**	-0.02	-0.02	-0.02	0.00	0.00
	**C3’**	0.00	0.00	0.00	0.00	0.00
	**C4’**	0.06	0.06	0.06	0.00	0.00
	**O4’**	-0.03	-0.03	-0.03	0.00	0.00
	**C5’**	0.07	0.07	0.07	0.00	0.00
	**O5’**	-0.04	-0.04	-0.04	0.00	0.00
	**C6’**	-0.02	-0.01	-0.02	0.00	0.00
**Phloroglucinol (A²)**	**C5**	-0.06	-0.01	-0.06	0.05	0.02
	**O5’**	0.09	0.10	0.09	0.02	0.01
	**C6**	-0.05	0.02	-0.05	0.06	0.03
	**C7**	0.08	0.14	0.08	0.05	0.03
	**O7’**	-0.04	0.01	-0.05	0.05	0.03
	**C8**	0.08	0.13	0.08	0.05	0.02
	**C9**	-0.03	0.02	-0.04	0.05	0.03
	**C10**	-0.04	0.02	-0.04	0.06	0.03
**Pyran (B²)**	**O1**	0.11	0.12	0.11	0.00	0.01
	**C2**	-0.13	-0.12	-0.14	0.00	0.01
	**C3**	0.13	0.13	0.11	-0.01	0.01
	**O3**	-0.20	-0.19	-0.20	0.01	0.00
	**C4**	0.02	0.04	0.02	0.02	0.01
**Catechol (C²)**	**C1’**	-0.03	-0.03	-0.05	-0.02	0.01
	**C2’**	0.00	0.01	-0.10	-0.10	0.05
	**C3’**	-0.01	-0.01	-0.06	-0.04	0.03
	**C4’**	0.07	0.08	0.03	-0.03	0.02
	**O4’**	-0.01	0.00	-0.10	-0.08	0.05
	**C5’**	0.06	0.07	0.03	-0.02	0.02
	**O5’**	-0.06	-0.06	-0.10	-0.03	0.02
	**C6’**	-0.08	-0.08	-0.12	-0.03	0.02
**Phloroglucinol (A³)**	**C5**	-0.05	-0.02	-0.05	0.03	0.02
	**O5**	0.09	0.10	0.09	0.01	0.00
	**C6**	-0.05	-0.02	-0.05	0.03	0.01
	**C7**	0.08	0.11	0.08	0.03	0.02

	**O7**	-0.04	-0.01	-0.04	0.03	0.02
	**C8**	0.08	0.11	0.08	0.03	0.02
	**C9**	-0.02	0.01	-0.03	0.03	0.02
	**C10**	-0.02	0.01	-0.03	0.03	0.02
**Pyran (B^3)**	**O1**	0.02	0.04	0.02	0.02	0.01
	**C2**	0.11	0.13	0.11	0.01	0.01
	**C3**	0.14	0.15	0.14	0.01	0.01
	**O3**	-0.13	-0.12	-0.14	0.01	0.01
	**C4**	-0.21	-0.20	-0.21	0.01	0.00
**Catechol (C^3)**	**C1’**	-0.03	-0.04	-0.03	0.00	0.00
	**C2’**	0.00	0.01	0.00	0.00	0.00
	**C3’**	0.00	0.00	-0.01	0.00	0.00
	**C4’**	0.07	0.07	0.07	0.01	0.00
	**O4’**	0.01	0.01	0.01	0.01	0.00
	**C5’**	0.07	0.07	0.07	0.01	0.00
	**O5’**	-0.06	-0.05	-0.06	0.00	0.00
	**C6’**	-0.02	-0.02	-0.02	0.00	0.00
**Phloroglucinol (A^4)**	**C5**	-0.05	-0.05	-0.05	0.00	0.00
	**O5**	0.09	0.09	0.09	0.00	0.00
	**C6**	-0.04	-0.03	-0.05	0.00	0.01
	**C7**	0.09	0.09	0.08	0.00	0.00
	**O7**	-0.04	-0.03	-0.05	0.00	0.01
	**C8**	0.08	0.08	0.08	0.00	0.00
	**C9**	-0.03	-0.02	-0.03	0.00	0.00
	**C10**	-0.05	-0.04	-0.05	0.00	0.00
**Pyran (B^4)**	**O1**	0.02	0.03	0.01	0.00	0.01
	**C2**	0.11	0.12	0.10	0.00	0.01
	**C3**	0.14	0.14	0.12	-0.02	0.01
	**O3**	-0.15	-0.15	-0.17	-0.02	0.01
	**C4**	-0.22	-0.22	-0.23	0.00	0.00
**Catechol (C^4)**	**C1’**	-0.03	-0.03	-0.06	-0.04	0.02
	**C2’**	0.00	0.00	-0.09	-0.09	0.04
	**C3’**	-0.01	-0.01	-0.06	-0.05	0.02
	**C4’**	0.07	0.07	0.04	-0.04	0.02
	**O4’**	0.01	0.01	-0.10	-0.11	0.06
	**C5’**	0.07	0.07	0.03	-0.04	0.02
	**O5’**	-0.01	0.00	-0.03	-0.03	0.01
	**C6’**	-0.02	-0.02	-0.06	-0.04	0.02

Table S2. The Electronic state Energy in Hartree and Dipole Moment (DM) in Debye calculated on M06-2x/aug-cc-pVTZ level of theory in condensed phase using the non-equilibrated and equilibrated solvent solute interaction.

EpiC: (-)-Epicatechin; PCB2: Procyanidin B2; PCC1: Procyanidin C1; CTA2: Cinnamtannin A2.

		Electronic state Energy in Hartree and Dipole Moment (DM) in Debay						
**Compound**		**Adiabatic Cation**	**Vertical Cation Equilibrated**	**Vertical Cation NON-Equilibrated**	**Neutral**	**Vertical Anion NON-Equilibrated**	**Vertical Anion Equilibrated**	**Adiabatic Anion**
**Flavan**	Energy	-654.994243	-654.985023	-654.946418	-655.223162	-655.221794	-655.257434	-655.261861
	DM	7.53	7.67	6.22	2.42	2.43	10.14	10.63
**EpiC**	Energy	-1031.174367	-1031.163593	-1031.125023	-1031.40029	-1031.400071	-1031.432636	-1031.44356
	DM	14.09	13.83	8.22	7.20	4.65	14.62	13.33
**PCB2**	Energy	-2061.387134	-2061.376585	-2061.346411	-2061.611337	-2061.612178	-2061.640572	-2061.63441
	DM	26.22	26.83	11.36	8.97	10.59	24.60	22.11
**PCC1**	Energy	-3091.595786	-3091.585509	-3091.557546	-3091.81934	-3091.822765	-3091.849554	-3091.861495
	DM	34.28	34.66	12.60	10.20	4.89	27.25	31.59
**CTA2**	Energy	-4121.810292	-4121.801431	-4121.775916	-4122.034957	-4122.03934	-4122.06	-4122.066002
	DM	23.61	21.39	19.94	13.90	9.29	31.84	39.77
